# The Effects of *FGF4* Retrogenes on Canine Morphology

**DOI:** 10.3390/genes13020325

**Published:** 2022-02-10

**Authors:** Danika Bannasch, Kevin Batcher, Fabienne Leuthard, Michael Bannasch, Petra Hug, Denis J. Marcellin-Little, Peter J. Dickinson, Michaela Drögemüller, Cord Drögemüller, Tosso Leeb

**Affiliations:** 1 Department of Population Health and Reproduction, University of California—Davis, Davis, CA 95616, USA; klbatcher@ucdavis.edu; 2 Institute of Genetics, Vetsuisse Faculty, University of Bern, 3001 Bern, Switzerland; fabileuthard@gmail.com (F.L.); petrahug@bluewin.ch (P.H.); michaela.droegemueller@vetsuisse.unibe.ch (M.D.); cord.droegemueller@vetsuisse.unibe.ch (C.D.); tosso.leeb@vetsuisse.unibe.ch (T.L.); 3 Veterinary Center for Clinical Trials, School of Veterinary Medicine, University of California—Davis, Davis, CA 95616, USA; mjbannasch@ucdavis.edu; 4 Department of Surgical and Radiological Sciences, University of California—Davis, Davis, CA 95616, USA; djmarcel@ucdavis.edu (D.J.M.-L.); pjdickinson@ucdavis.edu (P.J.D.)

**Keywords:** dwarfism, chondrodystrophy, chondrodysplasia, height, body size, conformation

## Abstract

Two *FGF4* retrogenes (*FGF4L1* on chromosome 18 and *FGF4L2* on chromosome 12) have been identified to cause dwarfism across many dog breeds. Some breeds are nearly homozygous for both retrogenes (e.g., Dachshunds) and others are homozygous for just one (e.g., Beagles and Scottish Terriers). Since most breeds do not segregate both of these retrogenes, it is challenging to evaluate their individual effects on long bone length and body size. We identified two dog breeds selected for hunting ability, the Alpine Dachsbracke and the Schweizer Niederlaufhund, that segregate both of these retrogenes. Using individual measurements of height at the shoulder, back length, head width, thorax depth and width, and thoracic limb measurements, we evaluated the combined effects of *FGF4* retrogenes within these breeds. We applied multivariable linear regression analysis to determine the effects of retrogene copy numbers on the measurements. Copy numbers of both retrogenes had significant effects reducing height at the shoulders and antebrachial length, with *FGF4L1* having a much greater effect than *FGF4L2*. *FGF4L1* alone influenced the degree of carpal valgus and *FGF4L2* alone increased head width. Neither retrogene had an effect on thorax width or depth. Selectively breeding dogs with *FGF4L1* and without *FGF4L2* would likely lead to a reduction in the *FGF4L2*-related risk of intervertebral disc herniation while maintaining the reduction in leg length resulting from *FGF4L1.*

## 1. Introduction

Dog breeds come in a wide variety of shapes and sizes. Dwarfism or skeletal dysplasia characterizes many dog breeds and was selected to slow the speed of hunting dogs, allow entry into small burrows to pursue game and to reduce overall size for companion dogs (www.fci.be and www.akc.org (accessed on 25 January 2021)). Two different *FGF4* retrogenes have been associated with dwarfism across dog breeds [1,2]. Interestingly, the most common form of human dwarfism is caused by reoccurring activating mutations in the *FGFR3* gene, which is one of the receptors for *FGF4* [3]. Retrogenes are functional, active intronless copies of the parent gene that integrate into chromosomal DNA through the action of endogenous LINE 1 retrotransposons [4]. The exact mechanism linking *FGF4* retrogene expression and skeletal dysplasia is not defined; limited data are available relating to temporal and spatial expression of *FGF4* retrogenes and although FGF4 protein overexpression acting through FGFR3 is an intriguing comparative model, FGF ligands are known to bind to multiple receptors [1,2,5]. We identified five additional *FGF4* retrogenes in canids leading to the corrected nomenclature of the retrogenes as L for “like”, followed by a number representing the order in which they were discovered [6].

One of the known canine *FGF4* retrogenes is located on chromosome 18 (*FGF4L1*) and is associated with a phenotype termed chondrodysplasia [2]. The other *FGF4* retrogene is located on chromosome 12 (*FGF4L2*) and has been associated with a phenotype previously described as chondrodystrophy [7]. Chondrodystrophy is characterized by both short legs and abnormal intervertebral discs [7]. Chondrodysplasia was defined as dogs having very short legs and is required by many breed standards [2]. Both loci have been identified in across breed association studies for height at the shoulders [8,9,10]. However, the direct effect of each retrogene on height or individual bone segments has not been evaluated.

The breed distribution of the two *FGF4* retrogenes suggests that the effects of the retrogenes are additive, since the breeds with the shortest legs, for example the Dachshunds and Corgis, have both retrogenes at high allele frequencies [1,11]. Since the majority of dog breed standards are very prescriptive about height, most breeds are unlikely to segregate alleles for height. This has limited the ability to detect the effects of these variants on height within breeds. Two breeds, the Alpine Dachsbracke (AD) and the Schweizer Niederlaufhund (SN), segregate different copy numbers of both *FGF4* retrogenes within each breed [11], likely reflecting breed standards, emphasizing hunting ability rather than a specifically defined absolute height. Height at the shoulders in the SN varies from 35–43 cm with an additional tolerance of 2 cm in males and in the AD 34–42 cm (http://www.fci.be (accessed on 25 January 2021)). We directly evaluated genotype phenotype correlations with height at the shoulder in both breeds and demonstrated that the effects of the two *FGF4* retrogenes additively decrease height. In the SN, we also evaluated the effect on additional specific morphologic measurements obtained from individual dogs. 

## 2. Materials and Methods

### 2.1. Sample and Data Collection

DNA samples, sex and height at the shoulder measurements for 111 dogs of the AD breed (58 males) were obtained from dog owners. DNA samples were submitted for genetic testing to the Vetsuisse Biobank at the University of Bern. DNA and morphological measurements from 36 SN (11 males) dogs were obtained by the investigators at gatherings of the official breed club in Switzerland. Procedures were approved by the “Cantonal Committee for Animal Experiments” (Canton of Bern; permits 48/13, 75/16 and 71/19) and the UC Davis Institutional Animal Care and Use Committee (Protocol #21190).

### 2.2. Measurements

Height at the shoulders was reported by owners of AD (Supplemental Table S1). All other measurements were collected by one author, DB, from SN over 1 year of age (Supplemental Table S2). Height at the shoulders was measured in cm with a level and movable arm. Aluminum body calipers were used to measure width of skull at the widest point, width of the thorax at the widest point and depth of the thorax at the greatest depth, length of the antebrachium from the olecranon process of the ulna to the palmar surface of the manus with the elbow and carpus held at a 90° angle, and length of the manus from the cranial surface of the antebrachium to the distal aspect of the third phalanx of digits 3 and 4, with the elbow and carpus held at a 90° angle. A tape measure was used to measure the length of the humerus (proximal aspect of the humeral greater tubercle to the olecranon process of the ulna), scapula (dorsal border to the proximal aspect of the humeral greater tubercle), back length (distal edge of the scapula to the proximal aspect of the ilium). A goniometer was used to measure carpal valgus, which was recorded as the angle of deviation [11]. Reproducibility of these measures was determined by comparing repeated measures taken of the same three dogs on three different days by the same observer. The coefficient of variation was calculated for each measurement for each dog and the mean percentage reported (Supplemental Table S3).

### 2.3. Genotyping

Genotyping for *FGF4L1* and *FGF4L2* was performed using a PCR based assay as previously described [1]. *FGF4L1* is the *FGF4* retrogene referred to in this manuscript as chromosome 18 *FGF4* and *FGFL2* is the *FGF4* retrogene referred to in this manuscript as chromosome 12 *FGF4*. In brief, three primer PCR is used to flank the novel insertion sites for *FGF4L1* and *FGF4L2* in separate PCR reactions. The third prime is an *FGF4* internal primer. Different product sizes are generated for the absence and presence of the retrogene insertion. 

### 2.4. Statistical Analysis

One dog had an injury that prevented thoracic limb measurements of the right limb. Other limb measurements and back length measurements from both the left and right were used across all genotype classes. Multivariable linear regression analysis was used to identify contributions of genotype to measurement using stats package in R version 4.1.1 [12]. Sex, *FGF4L1* genotype (zero copies, one copy, two copies) and *FGF4L2* genotype (zero copies, one copy, two copies) were independent variables. Reference categories for categorical variables were zero copies for retrogene genotypes and female sex. Univariable analyses were performed initially, and all independent variables were tested for inclusion in the multivariable model if either retrogene had Wald *p* < 0.2. Results were reported as differences in mean length cm or percent change and surrounding 95% confidence intervals (CI) (Supplemental Tables S4 and S5). *p* values were reported for the inclusion of each variable in the model. Carpal valgus was analyzed by rank ordering the measurements first. 

## 3. Results

### 3.1. Evaluation of Height at the Shoulder

**Figure 1:**
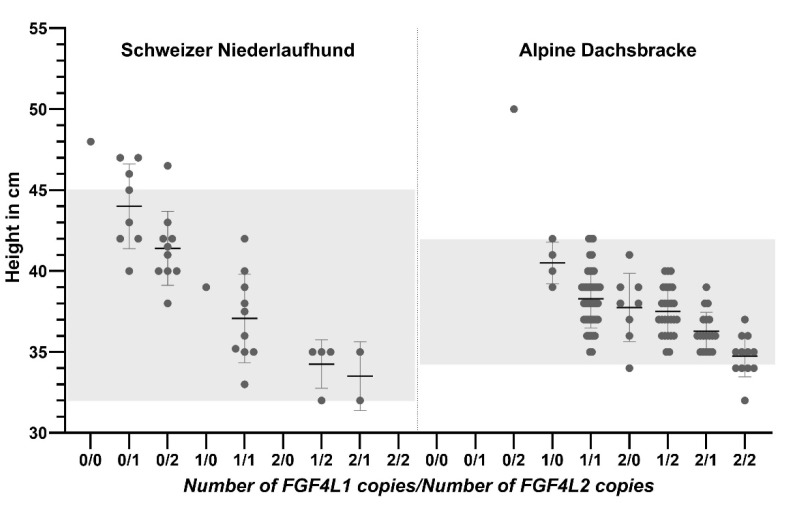
Height at the shoulder is shown for each genotype class for 36 Schweizer Niederlaufhund and 111 Alpine Dachsbracke dogs. The bars represent the mean and standard deviation for each genotype class. Some genotype classes did not have any dogs identified. The shaded areas show the allowed shoulder height according to breed standard for each breed. Genotype classes are ordered the same for both breeds and by descending means.

Two breeds of dogs which segregated different copy numbers of *FGF4* retrogenes were chosen to study the effects of the retrogenes on morphology. The Alpine Dachsbracke dogs (N = 111) had height at the shoulders reported by the owners and the Schweizer Niederlaufhund (N = 36) were measured during this study. Based on the possible combinations of the two retrogenes, there were nine possible genotype classes. However, not all genotype classes were represented in each breed (Figure 1). Based on comparing the mean height of each genotype class, there was an additive effect of copy number for each retrogene and between *FGF4L1* and *FGF4L2* on reducing height at the shoulder. In order to investigate this further, univariable and multivariable linear regression were performed to determine the effect of retrogene copy number and gender on height (Table 1 and Table 2). Copy numbers of both retrogenes and gender had significant effects on height at the shoulder. In both breeds, there was a high coefficient of multiple correlation between height and the number of copies of the *FGF4* retrogenes (r^2^ = 0.75 in SN and 0.71 in AD). The magnitude of the effect on height reduction was much greater for *FGF4L1* (15.9 to 27.9%) than for *FGF4L2* (3.2 to 10.5%) in both breeds.

### 3.2. Evaluation of Specific Morphologic Parameters in the Schweizer Niederlaufhund

**Figure 2:**
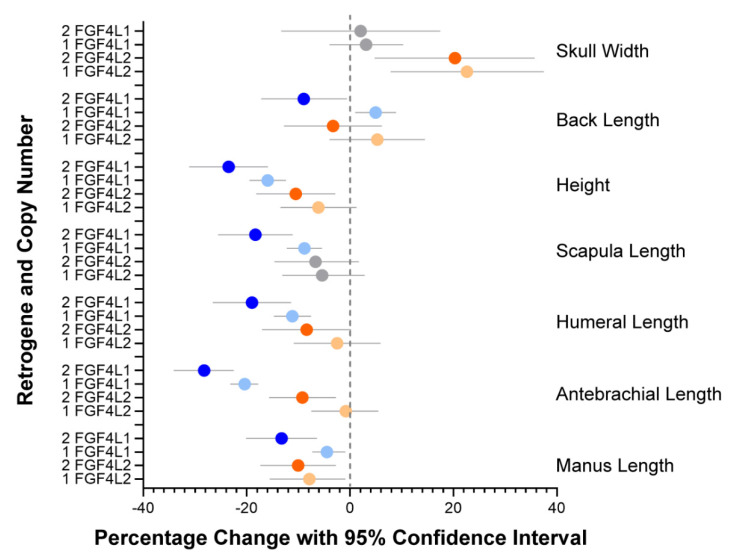
Measurements significantly affected by *FGF4* retrogenes. The mean percentage change and 95% confidence interval in multivariable analysis of the measurements is shown. The dotted line represents the value of 0 copies of either retrogene. Blue color represents statistically significant effects of FGF4L1, and orange color represents statistically significant effects of FGF4L2. Shades of blue and orange represent copy number. Grey symbols were not significant. Complete multivariable results are in Supplemental Table S5.

Measurements of thoracic limb segments, thorax depth and width, back length, head width and amplitude of carpal valgus were determined. The mean coefficient of variation ranged from 2.1% to 4.1% for the caliper measurements, 3.0–7.2% for the tape measurements and 2.3% for the shoulder height. Carpal valgus measured with a goniometer had a mean coefficient of variation of 14.5% (Supplemental Table S3). Univariable and multivariable linear regression were performed for all measurements to determine the effect of retrogene copy number and sex (Supplemental Tables S4 and S5). *FGF4L1* copy number was found to have a significant effect on increasing carpal valgus (*p* < 2 × 10^−16^) and reducing scapular length (*p* = 1.1 × 10^−7^). *FGF4L2* copy number was found to have a significant effect increasing head width (*p* = 0.015). Effects of copy number of both retrogenes were statistically significant, reducing humerus length (*FGF4L1 p* = 8.3 × 10^−9^, *FGF4L2 p* = 0.008), antebrachial length (*FGF4L1 p* = 2.0 × 10^−16^, *FGF4L2 p* = 1.6 × 10^−16^), and manus length (*FGF4L1 p* = 0.0002, *FGF4L2 p* = 0.03). The largest effect was on antebrachial length, where two copies of *FGF4L1* reduced length by 28% and two copies of *FGF4L2* reduced the length by 10%. Neither retrogene had a significant effect on thorax depth or width. A paradoxical effect for both retrogenes was seen on back length, where one copy of the retrogene increased back length and two copies decreased back length (*FGF4L1 p* = 0.001, *FGF4L2 p* = 0.0007). A summary of the significant results is shown in Figure 2. Sex had a significant effect on manus length (*p* = 0.005), scapular length (*p* = 0.04), and head width (*p* = 0.003).

## 4. Discussion

*FGF4* retrogenes had an additive effect reducing height at the shoulders in two breeds of dog. Individual thoracic limb segments performed in one breed were all affected by *FGF4L1*, but only the more distal segments showed significant effects of *FGF4L2*. Neither *FGF4* retrogene had an effect on thorax depth or width. *FGF4L1* alone affected carpal valgus, which is consistent with other breed phenotypes. For example, Beagles and American Cocker spaniels that only have *FGF4L2* do not typically have carpal valgus as a breed-related phenotype, while Basset hounds and Dachshunds do [13]. Interestingly, *FGF4L2* caused increasing skull width, which has not been reported previously but is also consistent with some breed traits, for example in French Bulldogs and Beagles [13].

Limitations of this study include using owner reported heights in the AD without the ability to assess the reproducibility of the measurements. In addition, the limited number of dogs of both sexes in each genotype class for the SN could have masked more subtle effects of both retrogenes on the measurements. Sex was always a variable used for the multivariable models and was significant for height, skull width, scapula, humeral and manus length.

Several other studies evaluated the effects of height-related alleles segregating within breeds. In the Labrador retriever, a *COL11A2* missense variant conferred a 10% reduction in height at the shoulders [14]. A variant in *ADAMTS17* confers a risk of glaucoma in Petit Basset Griffon Vendéen (PBGV) and Shar Pei but was also demonstrated to have an effect on height. In the Shar Pei, a 6% reduction in height was observed within homozygous animals and in the PBGV, a 4% reduction was observed [15]. The PBGV is a dwarf breed homozygous for *FGF4L1*. One study evaluated the effect of *FGF4L1* on height within the Havanese breed and identified that dogs homozygous for *FGF4L1* were 4.9 cm shorter than heterozygotes, with a 16% decrease in height [16]. In that study, only *FGF4L1* was evaluated, however *FGF4L2* also segregates in the breed [17].

Dwarfism in dogs has not been characterized relative to its effects on different long bone segments. Based on the data presented in this manuscript, the antebrachium is the most affected by *FGF4L1*, however all segments of the thoracic limb had a significant decrease in length. *FGF4L2* affected all segments except the scapula but to a lesser degree than *FGF4L1*. Both retrogenes had additive effects on the thoracic limb. The two described *FGF4* retrogene insertions lead to semi-dominant gain of function alleles [1,2]. The resulting phenotypic differences are most likely due to the specific expression control based on the insertion sites [1,2,6]. Further evidence for this is seen in the distinct phenotype differences seen in dogs with different *FGF4L1* and *FGF4L2* genotype combinations [16]. One interesting finding was the increase in head width significantly associated with *FGF4L2*, but not *FGF4L1*. Previous volumetric evaluation of canine skull based on CT scans identified *FGF4L1* as increasing the neurocranium in dogs [18]. These studies did not identify any association with *FGF4L2*, however our measurement was for the width of the skull rather than its volume. This phenotype could also be a driving selection for the *FGF4L2* insertion allele.

We identified a paradoxical relationship with both retrogenes and back length, where heterozygotes had longer backs and homozygotes had shorter backs than dogs without the retrogenes. The method that was used to measure back length was a tape from the distal scapula border to the proximal ilium. This paradoxical effect may have been caused by the presence of lordosis in dogs with two *FGF4* retrogene copies but not in dogs with one copy. There could also be additional genetic or environmental factors segregating in these dogs. Studies aimed at precisely measuring vertebral body length might be able to address this question more appropriately.

Breed height standards constrain the population of animals that may be chosen for breeding, which may maintain *FGF4L1* in the homozygous state since it has such a strong effect on height. Batcher et al. [11] identified eight breeds homozygous for *FGF4L1* and only three breeds homozygous for *FGF4L2*. *FGF4L2* has a more modest effect on height, particularly in the context of breeds homozygous for *FGF4L1*, allowing it to segregate in more breeds (37 vs. 24) [11]. One of the motivations for this study was to determine how strong the effect of *FGF4L2* is on the phenotypes of dogs, since *FGF4L2* is undesirable due to its association with Hansen type I intervertebral disc disease [11]. In the AD, being homozygous for *FGF4L2* reduces the shoulder height by only 6% and, in this breed, those dogs were right in the middle of the height standard. Reducing or eliminating *FGF4L2* from breeds due to its associated significant health concerns and its modest effect on height should be possible, particularly in breeds that segregate *FGF4L2*.

## Figures and Tables

**Table 1:** Multivariable analysis of height in cm at the shoulders in 111 Alpine Dachsbracke dogs.

	N	Mean	95% CI	Percent Change	*p*
*FGF4L1*					2.20 × 10^−16^
0 copies	1	50.4	47.7 to 53.1	-	
1 copy	72	38.7	33.4 to 43.9	−23.48%	6.1 × 10^−15^
2 copies	38	36.5	31.2 to 41.7	−27.90%	2.0 × 10^−16^
*FGF4L2*					1.7 × 10^−07^
0 copies	12	50.4	47.7 to 53.1	-	
1 copy	60	49.2	45.6 to 52.7	−3.21%	3.9 × 10^−03^
2 copies	39	48.0	44.5 to 51.6	−6.13%	3.1 × 10^−07^
Sex					1.64 × 10^−12^
Female	53	50.4	47.7 to 53.1	-	
Male	58	52.4	49.2 to 55.6	5.42%	

**Table 2:** Multivariable analysis of height at the shoulders in cm in 36 Schweizer Niederlaufhund dogs.

	N	Mean	95% CI	Percent Change	*p*
*FGF4L1*					2.7 × 10^−10^
0 copies	19	46.2	42.7 to 49.6	-	
1 copy	15	38.8	33.7 to 43.8	−15.9%	3.4 × 10^−10^
2 copies	2	35.3	28.4 to 42.2	−23.5%	6.1 × 10^−7^
*FGF4L2*					0.01
0 copies	2	46.2	42.7 to 49.6	-	
1 copy	20	43.3	36.3 to 50.1	−6.1%	0.10
2 copies	14	41.3	28.4 to 48.3	−10.5%	0.009
Sex					0.03
Female	25	46.2	42.7 to 49.6	-	
Male	11	48.3	42.9 to 53.5	4.5%	

## Data Availability

All data is available within the manuscript.

## Supplementary Materials

The following are available online at https://www.mdpi.com/article/10.3390/genes13020325/s1, Table S1. Raw data from the Alpine Dachsbracke breed, Table S2. Raw data from the Schweizer Niederlaufhund breed, Table S3. Coefficient of Variation for measurements of the Schweizer Niederlaufhund, Table S4. Multivariable Regression Analysis of the Alpine Dachsbracke, Table S5. Univariable and multivariable analysis of individuals measurements from the Schweizer Niederlaufhund.
